# Introducing *PLOS Biology*'s "Research Matters"

**DOI:** 10.1371/journal.pbio.1002518

**Published:** 2016-07-18

**Authors:** The PLOS Biology Editors

**Affiliations:** Public Library of Science, San Francisco, California, United States of America

## Abstract

*PLOS Biology*'s “Research Matters” is a new article series in which active scientists engage with the public about why basic research in their field matters. Research Matters will bridge the gap between researchers and the public by explaining how basic research positively impacts public health, society, life, and the environment.

In this issue of *PLOS Biology* we introduce a new magazine article type in which we invite active scientists across all of biology to share with the public why basic research in their field matters. We are pleased to announce “Research Matters” as the second new article type that we are rolling out this month after Open Highlights [1], developing our magazine section further. This new article type was inspired by the highly successful *PLOS Pathogens* Research Matters collection [2]. Given the broader remit of *PLOS Biology*, we can now expand the scope of this format to *PLOS Biology* to cover the rest of the life sciences (Fig 1).

**Fig 1:**
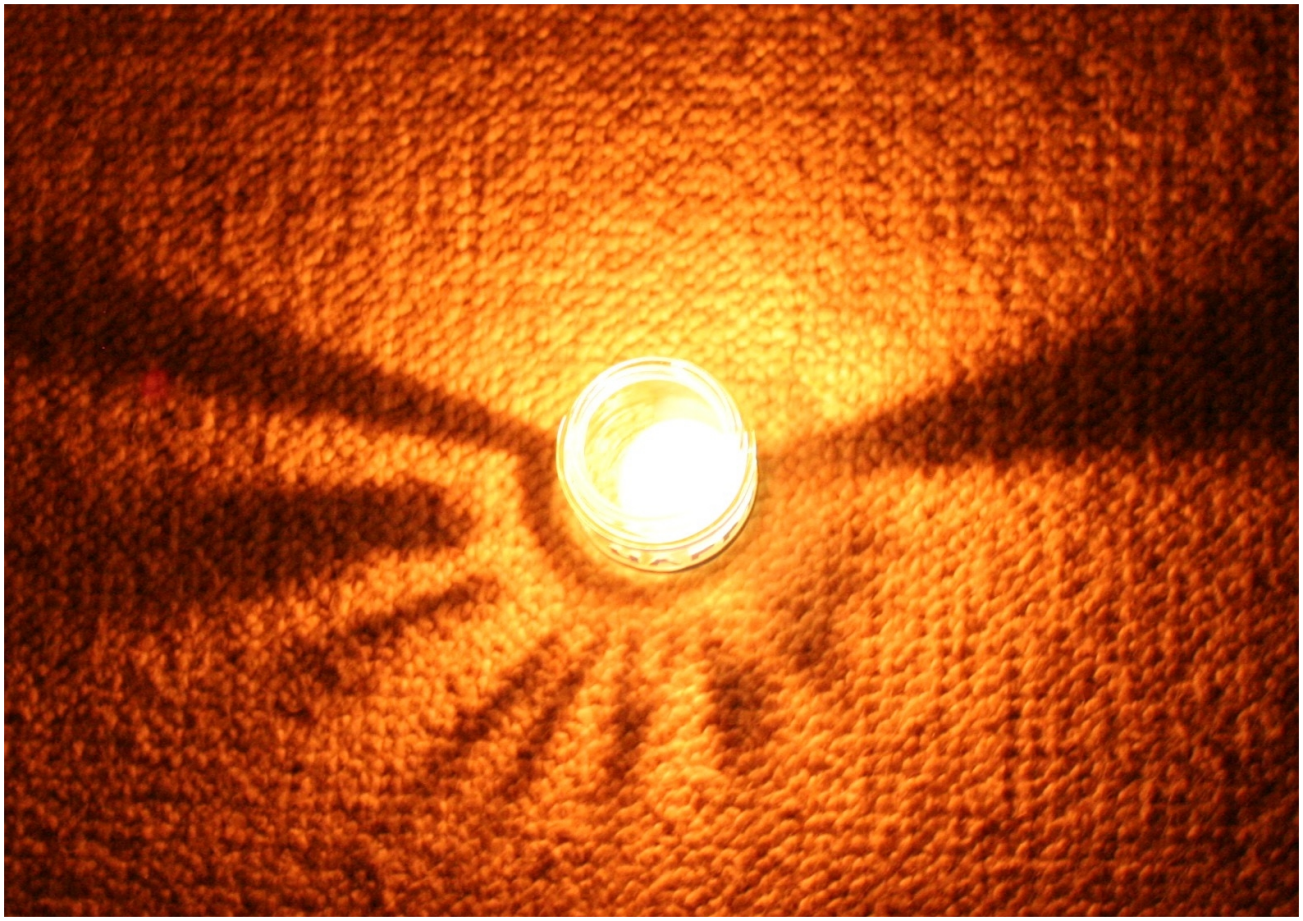
Research Matters. As part of our new series we will publish exciting pieces that explain why basic research matters to the general public. *Image credit*: *Anna Miska-Alvarez*.

Society has benefited from advances in science over the last two centuries, but it’s not always clear where the roots of all of these great discoveries lie. Just as you cannot start building a house with the roof, all research needs a basic foundation; yet the foundation for most impactful scientific discoveries are often unappreciated, or even unrecognizable, once the findings reach the general public.

The result is that there is often a disconnect between what the public perceives as the need behind basic research, and what the scientists carrying out the research aim to achieve. Our goal is to address this gap by enhancing the public’s understanding of the ultimate benefits of basic research to public health, society, life, and the environment. We believe that our audience and the general public will benefit greatly from hearing what ultimately drives scientists’ efforts in the lab.

In this series, we ask leading scientists in their respective fields to explain clearly and engagingly for a lay audience why the research carried out in their laboratories—and those of their collaborators and their colleagues—matters. Research Matters pieces should be informal and conversational and include anecdotal and autobiographical information. With this format we hope that the articles will be informative for scientists, institutions, funding bodies and the general public. We anticipate this series will also facilitate public engagement by researchers, allowing them to communicate the wide-reaching benefits arising from basic research carried out in a specific field.

We’re delighted to launch this new *PLOS Biology* series with a Research Matters piece from Michael Laub [3] in which he describes recent studies aimed to gain a basic understanding of how information is processed in biological systems. Laub’s discussion provides a window into how basic research into cell signalling can influence future treatment of bacterial infections and cancer. Upcoming pieces will build on this theme in communicating the diversity and impact of scientific advances that arise from basic research. We’ll be commissioning the majority of these Research Matters, but if you are interested in contributing to the series, please feel free to contact us.

As this new series grows in the journal, building upon the *PLOS Pathogens* series that came before it, we expect Research Matters articles will provide a compelling collection of narratives to illustrate the impact that fundamental research across biology has on human health and the world we live in, sometimes in unexpected ways.
